# Connectivity Patterns of Subthalamic Stimulation Influence Pain Outcomes in Parkinson's Disease

**DOI:** 10.3389/fneur.2020.00009

**Published:** 2020-02-12

**Authors:** Rubens Gisbert Cury, Manoel Jacobsen Teixeira, Ricardo Galhardoni, Valquiria Silva, Ricardo Iglesio, Carina França, Débora Arnaut, Erich Talamoni Fonoff, Egberto Reis Barbosa, Daniel Ciampi de Andrade

**Affiliations:** ^1^Department of Neurology, Movement Disorders Center, School of Medicine, University of São Paulo, São Paulo, Brazil; ^2^Neurosurgery Division, Department of Neurology, School of Medicine, University of São Paulo, São Paulo, Brazil; ^3^Transcranial Magnetic Stimulation Laboratory, Psychiatry Institute, University of São Paulo, São Paulo, Brazil; ^4^Department of Neurology, Pain Center, LIM 62, School of Medicine, University of São Paulo, São Paulo, Brazil; ^5^Pain Center, Instituto do Câncer do Estado de São Paulo, São Paulo, Brazil

**Keywords:** deep brain stimulation, connectivity, pain, sensibility, Parkinson's disease

## Abstract

**Background:** Pain is highly prevalent in Parkinson's disease and is associated with significant reduction in health-related quality of life. Subthalamic deep brain stimulation can produce significant pain relief in a subset of patients after surgery. However, the mechanism by which deep brain stimulation modulates sensory function in Parkinson's disease remains uncertain.

**Objective:** To describe the motor and pain outcomes of deep brain stimulation applied to a series of patients with Parkinson's disease and to determine whether the structural connectivity between the volume of tissue activated and different regions of the brain was associated with the changes of these outcomes after surgery.

**Methods:** Data from a long-term prospective cohort of 32 Parkinson's disease patients with subthalamic stimulation were combined with available human connectome to identify connections consistently associated with clinical improvement (Unified Parkinson Disease Rating Scale), pain intensity, and experimental cold pain threshold after surgery.

**Results:** The connectivity between the volume of tissue activated and a distributed network of sensory brain regions (prefrontal, insular and cingulate cortex, and postcentral gyrus) was inversely correlated with pain intensity improvement and reduced sensitivity to cold pain after surgery (*p* < 0.01). The connectivity strength with the supplementary motor area positively correlated with motor and pain threshold improvement (*p* < 0.05).

**Conclusions:** These data suggest that the pattern of the connectivity between the region stimulated and specific brain cortical area might be responsible, in part, for the successful control of motor and pain symptoms by subthalamic deep brain stimulation in Parkinson's disease.

## Introduction

Pain has a prevalence of 40–85% in Parkinson's disease (PD) patients (1, 2) and is related with a significant reduction in their quality of life (3). Subthalamic deep brain stimulation (STN DBS) is an effective treatment for the motor symptoms of PD (4), but it also ameliorates non-motor symptoms, such as pain (5). It has been shown that STN DBS can produce significant pain relief in more than 80% of PD patients and might be a major driver of quality of life improvement in the long term after DBS (5, 6). Besides pain intensity reduction, some studies have proposed that DBS can modulate conscious perception of sensory function, increasing the abnormally low sensory detection and pain thresholds seen in PD toward normal values (7–9). However, the mechanism by which DBS modulates sensory function in PD remains uncertain. Studies have failed to find a correlation between the amount of motor improvement and pain improvement after surgery, which is inconsistent with the musculogenic theory of pain in PD and suggests a more complex relationship among pain improvement, sensory changes, and motor improvement after surgery (10, 11).

Studies have demonstrated that the benefit of locally applied DBS to the STN might rely on the modulation of distant brain areas connected to the stimulation spot, through antidromic activation of neuronal somas, passing fibers, and afferent terminals from the cortex (12, 13). These remote influences of DBS can be measured by studying the fiber tracts that structurally connect both the volume of the tissue activated (VTA) and the corresponding distant area. Recently, the strength of the connectivity between the VTA and the supplementary motor area (SMA) was positively correlated with the motor response in a cohort of PD patients receiving STN DBS (14). This opens the fascinating possibility of tailoring the exact hot spot stimulation site to obtain clinical effects that are meaningful for the patient. However, while this possibility starts to sprout for the control of motor symptoms, no information exists concerning that “hot connectivity spot” related to non-motor symptom improvement after surgery, and pain in particular.

In PD, there is an abnormal functional overactivity in pain processing regions, such as the insula, the cingulate cortex, and the prefrontal cortex (8, 11), and fibers from these areas are known to reach the STN (15). In light of such a network-based mechanism of DBS action and motivated by our preceding connectivity study on motor symptoms, we explored the pain outcomes of DBS applied to a cohort of PD patients previously reported by our group (5, 9) in an attempt to determine whether the connectivity profile between a patient's DBS-VTAs and specific brain regions could correlate with pain intensity and thermic pain threshold changes after surgery, using an available human connectome data set (16).

## Materials and Methods

### Patients and Study Design

This study presents original imaging results from a previous clinical study on the effects of DBS on non-motor symptoms in PD (5, 9) In the present analyses, 32 patients with idiopathic PD according to the UKPD Society Brain Bank (17) who underwent STN DBS due to refractory motor complications were included. The patients had their motor (UPDRS-III) and pain scales prospectively evaluated before and 12 months after surgery. All implanted DBS electrodes were Medtronic 3387 (Minneapolis, MN, USA). This study was approved by our institution's ethics review board and registered in the clinical research database (# 0105/10). All patients were informed about the procedures in this protocol and gave informed consent to participate.

### Pain Assessment

Detailed protocol has been previously published (5, 9). Briefly, all participants underwent a quantitative sensory testing intended to assess temperature pain thresholds. The evaluations were performed before surgery in an off-medication condition and 12 months after surgery during off-medication/on-stimulation conditions. Tests were performed bilaterally on thenar eminences. A contact thermode was placed over the thenar eminence at a neutral temperature (32°C). Heat and cold pain thresholds (HPT, CPT) were assessed by the methods of limits (1°C/s change from 32°C). Temperatures were maintained within the range of 0–50°C to protect participants from thermal cutaneous injuries (9). Besides quantitative sensory testing analysis, all patients were classified as having pain directly related to PD (triggered by PD), i.e., pain temporally related to the disease course and that fluctuates according to the motor status and/or improves with antiparkinsonian drugs (18, 19). Non-parkinsonian-pain (pain related to etiologies other than PD) was not included. Pain intensity was measured with a 100-mm visual analog scale (0 = no pain, 100 = worst pain) (3) and the concerned patient's “pain in general.”

### Lead Location and Volume of Tissue Activated

Postoperative tomography was coregistered to preoperative T1- and T2-weighted MRI using SPM12 and then normalized into ICBM 2009b NLIN asymmetric space using the SyN method (http://stnava.github.io/ANTs/) (20). Brainshift was corrected when present. The DBS electrode contacts were located within Montreal Neurological Institute space using Lead-DBS software (http://www.lead-dbs.org) (21). Once the electrode was localized, the VTA of the active contact (cathode) was estimated using a heuristic stimulation algorithm previously described by Dembek et al. (22). The VTA was based on patient-specific stimulation parameters recorded 12 months after surgery. The overlap between the VTA and the STN was calculated in mm^3^. The sum of the two overlapping VTA/STN volumes from both hemispheres was correlated with the percent change in VAS and cold pain threshold in order to analyze whether the area stimulated inside the STN could influence pain outcomes.

### Connectivity Analysis

Using VTAs as seed regions, structural connectivity estimates were analyzed using a normative structural connectome, which consists of high-density normative fibertracts based on 20 subjects (16). Global fiber-tracking was performed using Gibb's tracking method (23). Structural connectivity was calculated by extracting tracts passing through VTA and calculating the fiber counts in a voxel-wise fashion in specific brain areas (16). Brain parcellations were defined according to the human Harvard-Oxford atlas, a probabilistic atlas covering 48 cortical and 21 subcortical structural areas, derived from structural data and segmentations (24). For pain correlation, we included pairs of sensory regions of interest related to classic pain circuitry and previously reported to be affected in PD (25–27): prefrontal cortex, insular cortex, cingulate gyrus anterior division, and post central gyrus (Supplementary Figure 1) (8, 28). Finally, for pain and DBS motor response, we also analyzed the correlation of VTA with the SMA, previously associated with the improvement of motor symptoms in PD (14) but also linked with pain modulation (discussed below).

### Statistical Analysis

Motor function (UPDRS-III), pain intensity (VAS), and sensory thresholds (HPT and CPT) were expressed as average ± standard deviation. Because the Kolmogorov–Smirnov test disclosed that the values did not have a normal distribution, Wilcoxon signed rank test was applied. Spearman coefficients were used to assess the variables' correlations. The level of statistical significance was set at *p* < 0.05 and was then lowered according to the Bonferroni correction for multiple comparisons (for VAS, the *p* value was set at <0.005 and for pain thresholds at <0.01).

The connectivity from VTAs was calculated for each patient. Structural connectivity strength was defined as the number of fiber tracts between VTA and the corresponding cortical area. This procedure resulted in R-maps with Spearman's rank-correlation coefficients for each voxel. The independent variable was defined as the VAS change (expressed in %), CPT change (CPT before–after), and DBS motor response (UPDRS-III in the off-medication before surgery–UPDRS-III off-medication/on-stimulation) 12 months after surgery. We did not include in the analysis the HPT because it did not change after the surgery.

## Results

All 32 patients were included in the analysis. The mean duration of the disease was 15.4 ± 8.1 years, and the Hoehn & Yahr off-medication score was 2.7 ± 0.6. Preoperative UPDRS-III scores were 45.1 ± 12.3 in the off-medication and 16.8 ± 7.6 in the on-medication conditions. After STN DBS, the UPDRS-III scores in the off-stimulation/off-medication condition were 46.9 ± 13.4 and 23.9 ± 10.6 in the on-stimulation/off-medication condition (49% of improvement).

### Pain Outcomes

Twenty-three patients (71.9%) had pain related to PD before the surgery. After STN DBS, eight patients (28.1%) remained with pain under their regular pharmacological treatment (*p* < 0.001). In those who remained symptomatic, there was significant reduction in pain intensity after surgery (VAS: before = 66.0 ± 24.1, after *r* = 42.5 ± 19.0, *p* = 0.011). One patient developed dystonic pain after surgery in the left arm. Comparing to baseline, STN DBS significantly decreased the CPT (reduced sensitivity to cold pain) after surgery in both hands (left side before = 18.4 ± 7.8, after = 13.0 ± 8.4; right side before = 18.1 ± 7.0, after = 10.3 ± 6.4; *p* = 0.007 and 0.003, respectively). There were no changes in the HPT after surgery (left side before = 41.1 ± 5.1, after = 41.7 ± 4.7; right side before = 41.4 ± 4.4, after = 42.0 ± 5.6, *p* > 0.05). No correlation was found between the change in pain intensity (VAS) and the CPT (left side, *r* = 0.221, *p* = 0.800; right side, *r* = 0.114, *p* = 0.123) and between VAS and the CPT with motor response to STN DBS (*p* > 0.05).

Once it was determined that both VAS and the CPT changed after surgery, but did not correlate with each other, imaging analysis was performed based on these two variables in order to determine whether the STN volume was stimulated and the connectivity pattern between the VTA and sensory cortical areas could account for these changes.

### Contact Position and Imaging Analysis

**Figure 1:**
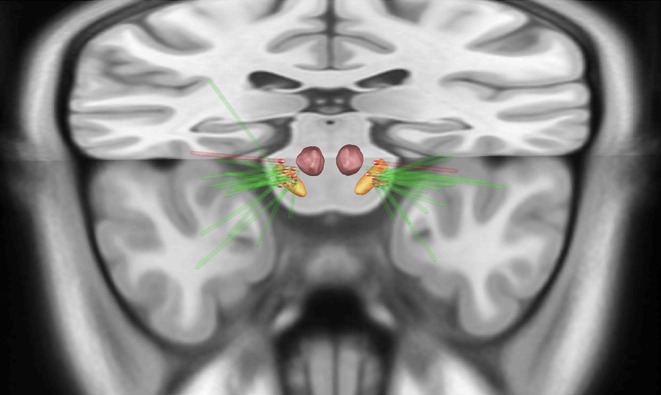
Upper view of the lead placement and the active contact (red highlighted) from all patients with pain before surgery (green electrodes) localized in the subthalamic nucleus (STN). The red electrode represents the patient who developed painful dystonia after surgery, showing the contact posteriorly to the STN. Orange = STN, red = red nucleus.

For both sides, the ventral contacts were the most frequently utilized as cathode (Table 1 shows the contacts and the parameters applied in each brain sides). Spatially, most contacts were located in the dorsal part of the STN (Figure 1 and Supplementary Figure 2 illustrate the electrode position). The patient with *de novo* pain after surgery had the electrodes set posteriorly (Figure 1).

**Table 1:** Cathode distribution and parameters applied in each subthalamic nucleus at 12 months after surgery.

	**Most ventral (–)**	**Ventral (–)**	**Dorsal (–)**	**Most dorsal (–)**	**Current (mA)**	**Pulse width (μs)**	**Frequency (Hz)**
Left STN	12	15	04	01	2.5 ± 0.5	76.8 ± 17.4	131.8 ± 23.3
Right STN	11	17	13	01	2.6 ± 0.5	80.6 ± 20.7	131.8 ± 23.3

*STN, subthalamic nucleus*.

**Figure 2:**
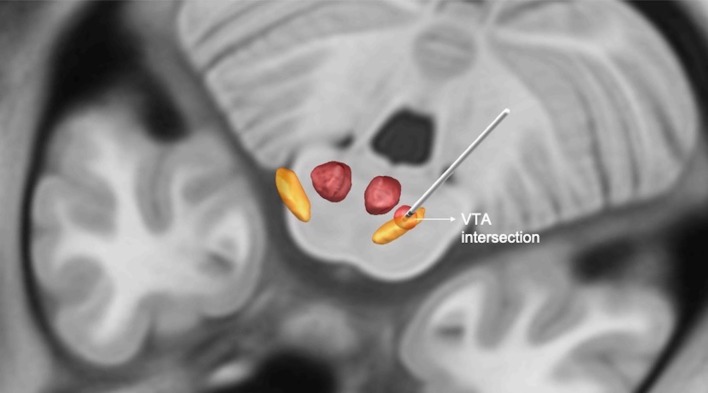
Illustration of the intersection between the volume of tissue activated (VAT) and subthalamic nucleus (STN) in mm^3^. Orange = STN, red = red nucleus, circumferential red circle around the electrode = VTA.

**Figure 3:**
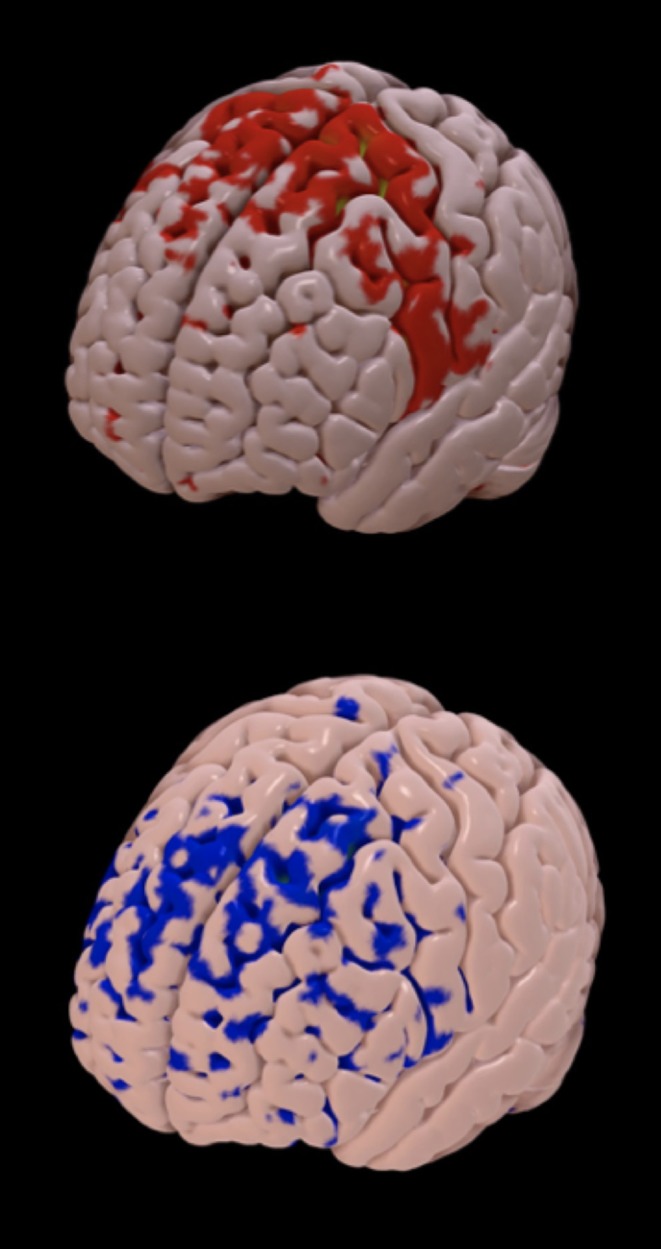
Structural connectivity between the volume of tissue activated and cortical areas. The brain map represents the cortical areas structurally connected with the volume of tissue activated from a good responder (improvement in pain intensity and cold pain threshold after surgery; red areas, mainly located in the supplementary motor area) and a poor responder (blue areas, mainly located anteriorly in the frontal lobe).

There was no relation between the VTA intersection of the STN with VAS (*n* = 23, *p* = 0.174) or CPT changes (*n* = 32, *p* = 0.362) after surgery (Figure 2). Using the structural connectivity between the VTAs and cortical areas described above, we identified that the left prefrontal cortex (*r* = −0.528, *p* = 0.001) and the right post-central gyrus (*r* = −0.323, *p* = 0.004) correlated negatively with VAS improvement (*n* = 23). Additionally, the right prefrontal cortex (*r* = −0.517; *p* = 0.008) correlated negatively with left CPT improvement (n=32). The left prefrontal cortex (*r* = −0.666; *p* = 0.002), the left insular cortex (*r* = −0.548; *p* = 0.003), and the left cingulate gyrus anterior division (*r* = −0.547; *p* = 0.003) correlated negatively with right CPT reduction, whereas there was a strong positive correlation with the left SMA (*r* = 0.676; *p* = 0.002) (Figure 3). Finally, there was a positive correlation between the DBS motor response with the left SMA (*r* = 0.404; *p* = 0.011).

## Discussion

Our primary findings were that: (i) STN DBS alleviates pain intensity and reduces CPT 12 months after surgery in PD, but these changes differed between patients and were not correlated with each other; (ii) the VTA inside the STN does not explain the variance in pain change after surgery; and (iii) the pattern of the connectivity between the stimulated region and specific brain cortical areas may be responsible for this variance in outcome. This latter point reinforces the recent, growing evidence that, although the targets for DBS in neurological disorders are normally determined by specific anatomical regions (e.g., nucleus or tracts), the ideal target may not necessarily be an anatomical structure itself but rather a structurally connected region. This has an obvious surgical targeting implication, but also highlights the importance of postoperative symptom-oriented programming in looking for the desired network within the same structural DBS target.

It is well-known that STN DBS not only produces motor improvements but also influences a set of non-motor symptoms, including pain related to PD (5). In addition to pain relief, STN DBS has also been associated with improved sensory detection and pain thresholds, which are modified toward normal values after surgery (7–9, 29). What is uncertain is whether the remote effect of DBS mediated through structural connectivity could account for those non-motor symptom changes or whether those changes are due to a global improvement in motor function after surgery. Accordingly, the current study utilized a DBS cohort (*n* = 32) to explore the relationship between their sensory changes and the structural connectivity of the VTA with specific brain regions. We have found that the brain regions responsible for central pain processing (11) were negatively correlated with the effectiveness of STN DBS in ameliorating pain intensity and induced cold pain, meaning that the higher the influence of the STN on these areas, the lower the improvement in spontaneous or induced pain after surgery. Interestingly, STN DBS affects more the CPT than the HPT, probably reflecting the fact that the HPT is usually less affected in PD patients (9). Additionally, there are qualitative differences between thermal pain thresholds. CPTs are highly modulated by top-down systems, such as the opioidergic and cannabinoid ones, known to be influenced by DBS (30, 31). Also, the CPT and the HPT are differentially conveyed to the SNC, with the CPT being mediated by C and A delta fibers, while the HPT being mainly dependent on unmyelinated C fibers (32).

A functional study demonstrated, in PD, an abnormal pain-induced activation in both sensory discriminative processing of pain, as occurs in the insula, in affective motivational processing of pain, as occurs in the cingulate cortex, and in cognitive areas, such as the prefrontal cortex (28). In addition, levodopa reduced pain-induced activation in those same overactive sensory areas. Remarkably, we have recently shown in a different model (central neuropathic pain), where the insula and anterior cingulate cortex are known to be hyperactive, that non-invasive deep transcranial magnetic stimulation of these structures not only was ineffective in alleviating pain but also actually aggravated some aspects of pain, especially in the insula group (33).

The STN is a small nucleus that projects fibers to both pallidal divisions and to the substantia nigra and uses the excitatory neurotransmitter glutamate to mediate its function (34). In PD, the loss of GPe inhibition over the STN culminates with high-level activity in this nucleus, leading to the distinctive motor impairments seen in PD. The delivery of high-frequency electrical impulses to the dorsolateral STN through DBS interferes with the function of the STN and reduces its output, alleviating the symptoms (orthodromic effect). In addition to the decrement in STN output, DBS exerts its activity by modulating passing fibers and afferent terminals, including those from the cortex (antidromic effect). The stimulation of incoming fibers could antidromically activate several cortical areas in a retrograde manner, leading to widespread and heterogeneous effects at distal sites (13). Along this line, the question raised by the present study is whether the local stimulation of these different fibers that project to the STN could influence pain outcomes, particularly those fibers coming from sensory areas.

Most of the cortical afferents to the STN arise from the primary motor cortex, SMA, and the dorsal and ventral premotor cortex and predominantly innervate the dorsal aspects of the nucleus (35). The limbic ventromedial portion of the STN receives fibers from the prelimbic-medial orbital areas of the prefrontal cortex (36). Somatosensory projections from the cingulate cortex, somatosensory cortex, and insular cortex also primarily project to the medial part of the STN, but a specific somatotopy organization has not been described (37–40). We have shown an anticorrelation between the activation of these fibers and the improvement of pain after surgery. It is worth highlighting that, overall, the patients experienced improved pain intensity (except for one patient whose electrodes were located posteriorly) and reduced CPT after surgery, but the degree of this improvement among the patients was quite variable. We hypothesized that avoiding stimulating sensory/pain-related afferents to the STN seems to be reasonable in patients with moderate/severe pain related to PD at baseline in order to optimize the results. Because most of the sensory afferents enter in the medial part of the STN, we hypothesize that the ideal VTA should be located more laterally.

The present study was not intended or designed to find the stimulation “hot spot” for pain improvement. The STN DBS in PD probably modulates not only the nucleus itself but also a brain network of converging neural pathways from different brain regions as well as the nucleus outputs. It will probably be very difficult to define a specific area of stimulation that is ideal for improving both the motor and non-motor symptoms because it is impossible to disentangle them with certainty, which highlights the importance of personalized polarity and parameter trials in each electrode based on individual responses and symptoms.

In line with a previous study where stronger connectivity between the site of stimulation and the SMA was associated with better motor improvement in PD patients (14), we found a positive connection between the VTA and the SMA in terms of the DBS motor response. Interestingly, the VTA–SMA connectivity was also correlated with the CPT change after surgery. Although we found that motor and non-motor (CPT) changes may be correlated with the same brain area, previous studies showed that there are no correlations between pain and motor improvement after STN DBS (5, 11, 29). The changes in pain and sensory thresholds after STN probably are a patchwork of motor improvement and changes in motivation drive, in the capacity of patients to perform more physical activity, central mechanisms, and connectivity pattern (11).

Clinical and preclinical studies show that motor cortex stimulation induces analgesia by activating descending analgesic pathways (41). Motor cortex stimulation improves the nociceptive threshold in rats via endogenous opioids, inhibiting thalamic nuclei and activating the periaqueductal gray (42). Additionally, electrophysiology and functional imaging studies have shown that motor cortex stimulation activates the brain regions involved in the perception and/or emotional aspects of pain, including the lateral thalamus, anterior cingulate cortex, anterior insula, and periaqueductal gray (43). Interestingly, it has been repetitively shown that precentral gyrus stimulation by transcranial magnetic stimulation preferentially affects the CPT toward analgesia (44) and that this effect is dependent on endogenous opioids (45) and the availability of N-methyl-D-aspartate receptors (46). This suggests that CPT changes may occur due to motor/premotor back-stimulation and possibly serve as a marker of the stimulation that is delivered to more dorsal-motor-related areas instead of more medial-sensory/affective regions. Even so, because there is no correlation between changes in motor and non-motor symptoms after STN DBS, including pain and the CPT (5, 11), this assumption should be interpreted with caution and clearly remains to be determined in further studies.

In clinical practice, exploring the individual connectivity profile between the chosen cathodes and corresponding activated cortical regions would imply a more effective therapy based on the patient's motor and non-motor baseline status. For instance, in patients with significant pain related to PD at baseline, the cathode with higher SMA and lower sensory area connectivity would be the best option. Along this line, considering the new directional devices with more cathodes and consequently more VTA options, more personalized programming based on the patient's connectivity profile could bring better clinical results.

Our study has several limitations. First, the brain regions correlated with the VTA varied between the pain dimensions analyzed (VAS vs. pain thresholds) and between the sides (right and left), which limits us from drawing a more robust conclusion. In addition, due to the low alpha value after Bonferroni correction, many important correlations should be missed (type II error). Therefore, connectivity patterns that look at pain and other non-motor symptoms should be explored in larger studies. Second, the present study did not use a patient-based connectome, which would be preferable and more reliable, considering the possible anatomic variances in PD patients. Therefore, future studies using patients' connectomes should be performed to confirm our data. On the other hand, normative connectomes from healthy subjects have the benefit of large participant numbers, high-quality signal-to-noise ratios, and acquisition that involves operating unique high-power MRI scanners that are particularly designed for connectivity imaging. The connectome used in our study was created using the Gibbs global tracking algorithm (16), which has the advantage of reconstructing multiple fiber tracts passing through the ROIs and VTAs we analyzed. The method is very computationally consuming compared to other deterministic algorithms and, although the fact that we did not use the DTI data of each patient is a limitation to the study, it allowed us to identify interesting structural relations among different brain regions in this exploratory work. Moreover, a recent study evaluated the connectivity between the VTA and brain regions through both normative connectome from healthy subjects and a connectome that was age, sex, and disease matched (PD) (14). Their connectivity results (to predict motor outcome after STN DBS) were highly correlated across patients using the normative vs. PD connectome.

We decided to use a broad brain map parcellation, which spans large parts of the cortex. Subsequently, a more specific brain area connected to the VTA could not be identified, and further studies using brain parcellation with smaller and more specific cortical areas would be helpful in this issue. Finally, another important point concerns the current models of calculating the VTA. The models assume that the whole VTA is activating the tissue, but, instead, the VTA represents the volume of the electrostatic field, where the axons and cell bodies receive electrons but may or may not be activated. This limitation is intrinsic to the VTA models and should be refined in future studies.

Taking our findings and the literature review together, we can summarize that PD patients have higher pain prevalence and abnormal pain thresholds compared to controls and that the supraspinal areas involved in the nociceptive process are, overall, overactivated. Deep brain stimulation improves pain intensity and decreases sensitivity to cold pain and, in part, the amount of this change occurs through antidromic activation of the SMA (an area related to analgesia) and is associated with the avoidance of activation of subcortical/cortical sensory circuitry areas. Metabolic, electrophysiologic, and functional studies on this matter could confirm our preliminary findings. Further clinical studies are necessary to define how to work together with the strengths of normative connectomes and connectivity data from individual patients.

## Data Availability Statement

Anonymized data are available and will be shared upon reasonable request from any qualified investigator.

## Ethics Statement

This study was approved by our institution's ethics review board and registered in the clinical research database (# 0105/10). Ethical Committee: Comissão de Ética para Análise de Projetos de Pesquisa do HCFMUSP, Address: Rua Ovídio Pires de Campos, 225 – 5° andar – Prédio da Administração, Phone: 55 11 26 61 75 85, e-mail: cappesq.adm@hc.fm.usp.br. The patients/participants provided their written informed consent to participate in this study.

## Author Contributions

RC: conception, organization, and execution of Research Project, execution and data analysis of Clinical Assessments, writing of the first draft and review and critique of Letter. MT, RG, VS, RI, and EB: organization of Research Project, execution of Clinical Assessments, review and critique of Letter. CF, DA, EF, and EB: organization and execution of Research Project, execution of Clinical Assessments, review and critique of Letter. DC: conception, execution, and supervision of Research Project, data analysis of Clinical Assessments, review and critique of Letter.

### Conflict of Interest

The authors declare that the research was conducted in the absence of any commercial or financial relationships that could be construed as a potential conflict of interest.

## References

[1] Beiske AG Loge JH Rønningen A Svensson E. Pain in Parkinson's disease: prevalence and characteristics. Pain. (2009) 141:173–7. 10.1016/j.pain.2008.12.004 19100686

[2] Broen MPG Braaksma MM Patijn J Weber WEJ. Prevalence of pain in Parkinson's disease: a systematic review using the modified QUADAS tool. Mov Disord. (2012) 27:480–4. 10.1002/mds.24054 22231908

[3] Quittenbaum BH Grahn B. Quality of life and pain in Parkinson's disease: a controlled cross-sectional study. Parkinsonism Relat Disord. (2004) 10:129–36. 10.1016/j.parkreldis.2003.12.001 15036166

[4] Krack P Batir A Van Blercom N Chabardes S Fraix V Ardouin C. Five-year follow-up of bilateral stimulation of the subthalamic nucleus in advanced Parkinson's disease. N Engl J Med. (2003) 349:1925–34. 10.1056/NEJMoa035275 14614167

[5] Cury RG Galhardoni R Fonoff ET Dos Santos Ghilardi MG Fonoff F Arnaut D. Effects of deep brain stimulation on pain and other nonmotor symptoms in Parkinson disease. Neurology. (2014) 83:1403–9. 10.1212/WNL.0000000000000887 25217059

[6] Jung YJ Kim H-J Jeon BS Park H Lee W-W Paek SH. An 8-year follow-up on the effect of subthalamic nucleus deep brain stimulation on pain in Parkinson disease. JAMA Neurol. (2015) 72:504–10. 10.1001/jamaneurol.2015.8 25799451

[7] Ciampi de Andrade D Lefaucheur J-P Galhardoni R Ferreira KSL Brandão Paiva AR Bor-Seng-Shu E. Subthalamic deep brain stimulation modulates small fiber-dependent sensory thresholds in Parkinson's disease. Pain. (2012) 153:1107–13. 10.1016/j.pain.2012.02.016 22456108

[8] Dellapina E Ory-Magne F Regragui W Thalamas C Lazorthes Y Rascol O. Effect of subthalamic deep brain stimulation on pain in Parkinson's disease. Pain. (2012) 153:2267–73. 10.1016/j.pain.2012.07.026 22964434

[9] Cury RG Galhardoni R Teixeira MJ Dos Santos Ghilardi MG Silva V Myczkowski ML. Subthalamic deep brain stimulation modulates conscious perception of sensory function in Parkinson's disease. Pain. (2016) 157:2758–65. 10.1097/j.pain.0000000000000697 27559833

[10] Spielberger S Wolf E Kress M Seppi K Poewe W. The influence of deep brain stimulation on pain perception in Parkinson's disease. Mov Disord. (2011) 26:1367–8; author reply 1368–9. 10.1002/mds.23570 21400607

[11] Cury RG Galhardoni R Fonoff ET Perez Lloret S Dos Santos Ghilardi MG Barbosa ER. Sensory abnormalities and pain in Parkinson disease and its modulation by treatment of motor symptoms. Eur J Pain. (2016) 20:151–65. 10.1002/ejp.745 26147660

[12] Henderson JM. “Connectomic surgery”: diffusion tensor imaging (DTI) tractography as a targeting modality for surgical modulation of neural networks. Front Integr Neurosci. (2012) 6:15. 10.3389/fnint.2012.00015 22536176 PMC3334531

[13] Li Q Qian Z-M Arbuthnott GW Ke Y Yung W-H. Cortical effects of deep brain stimulation: implications for pathogenesis and treatment of Parkinson disease. JAMA Neurol. (2014) 71:100–3. 10.1001/jamaneurol.2013.4221 24189904

[14] Horn A Reich M Vorwerk J Li N Wenzel G Fang Q. Connectivity Predicts deep brain stimulation outcome in Parkinson disease. Ann Neurol. (2017) 82:67–78. 10.1002/ana.24974 28586141 PMC5880678

[15] Hamani C Saint-Cyr JA Fraser J Kaplitt M Lozano AM. The subthalamic nucleus in the context of movement disorders. Brain. (2004) 127:4–20. 10.1093/brain/awh029 14607789

[16] Horn A Ostwald D Reisert M Blankenburg F. The structural-functional connectome and the default mode network of the human brain. Neuroimage. (2014) 102:142–51. 10.1016/j.neuroimage.2013.09.069 24099851

[17] Hughes AJ Daniel SE Kilford L Lees AJ. Accuracy of clinical diagnosis of idiopathic Parkinson's disease: a clinico-pathological study of 100 cases. J Neurol Neurosurg Psychiatry. (1992) 55:181–4. 10.1136/jnnp.55.3.181 1564476 PMC1014720

[18] Ford B. Pain in Parkinson's disease. Mov Disord. (2010) 25 (Suppl. 1):S98–103. 10.1002/mds.22716 20187254

[19] Mylius V de Andrade DC Cury RG. Pain in Parkinson's disease: current concepts and a new diagnostic algorithm. Mov Disord Clin Practice. (2015) 2:357–64. 10.1002/mdc3.12217 30363602 PMC6178768

[20] Avants BB Epstein CL Grossman M Gee JC. Symmetric diffeomorphic image registration with cross-correlation: evaluating automated labeling of elderly and neurodegenerative brain. Med Image Anal. (2008) 12:26–41. 10.1016/j.media.2007.06.004 17659998 PMC2276735

[21] Horn A Kühn AA. Lead-DBS: a toolbox for deep brain stimulation electrode localizations and visualizations. Neuroimage. (2015) 107:127–35. 10.1016/j.neuroimage.2014.12.002 25498389

[22] Dembek TA Barbe MT Åström M Hoevels M Visser-Vandewalle V Fink GR. Probabilistic mapping of deep brain stimulation effects in essential tremor. Neuroimage Clin. (2017) 13:164–73. 10.1016/j.nicl.2016.11.019 27981031 PMC5144752

[23] Reisert M Mader I Anastasopoulos C Weigel M Schnell S Kiselev V. Global fiber reconstruction becomes practical. Neuroimage. (2011) 54:955–62. 10.1016/j.neuroimage.2010.09.016 20854913

[24] Makris N Goldstein JM Kennedy D Hodge SM Caviness VS Faraone SV. Decreased volume of left and total anterior insular lobule in schizophrenia. Schizophr Res. (2006) 83:155–71. 10.1016/j.schres.2005.11.020 16448806

[25] Peyron R Laurent B García-Larrea L. Functional imaging of brain responses to pain. A review and meta-analysis. (2000). Neurophysiol Clin. (2000) 30:263–88. 10.1016/S0987-7053(00)00227-6 11126640

[26] Hudson AJ. Pain perception and response: central nervous system mechanisms. Can J Neurol Sci. (2000) 27:2–16. 10.1017/S0317167100051908 10676581

[27] Davis KD. The neural circuitry of pain as explored with functional MRI. Neurol Res. (2000) 22:313–7. 10.1080/01616412.2000.11740676 10769826

[28] Brefel-Courbon C Payoux P Thalamas C Ory F Quelven I Chollet F. Effect of levodopa on pain threshold in Parkinson's disease: a clinical and positron emission tomography study. Mov Disord. (2005) 20:1557–63. 10.1002/mds.20629 16078219

[29] Marques A Chassin O Morand D Pereira B Debilly B Derost P. Central pain modulation after subthalamic nucleus stimulation: a crossover randomized trial. Neurology. (2013) 81:633–40. 10.1212/WNL.0b013e3182a08d00 23864314

[30] Ossipov MH Dussor GO Porreca F. Central modulation of pain. J Clin Invest. (2010) 120:3779–87. 10.1172/JCI43766 21041960 PMC2964993

[31] DosSantos MF Oliveira AT Ferreira NR Carvalho ACP Rosado de Castro PH. The contribution of endogenous modulatory systems to TMS- and tDCS-induced analgesia: evidence from PET studies. Pain Res Manag. (2018) 2018:2368386. 10.1155/2018/2368386 30538794 PMC6257907

[32] Yarnitsky D Sprecher E Zaslansky R Hemli JA. Heat pain thresholds: normative data and repeatability. Pain. (1995) 60:329–32. 10.1016/0304-3959(94)00132-X 7596629

[33] Galhardoni R Aparecida da Silva V García-Larrea L Dale C Baptista AF Barbosa LM. Insular and anterior cingulate cortex deep stimulation for central neuropathic pain: disassembling the percept of pain. Neurology. (2019) 92:e2165–75. 10.1212/WNL.0000000000007396 30952795

[34] Mavridis I Boviatsis E Anagnostopoulou S. Anatomy of the human subthalamic nucleus: a combined morphometric study. Anat Res Int. (2013) 2013:319710. 10.1155/2013/319710 24416591 PMC3876692

[35] Nambu A Tokuno H Takada M. Functional significance of the cortico-subthalamo-pallidal “hyperdirect” pathway. Neurosci Res. (2002) 43:111–7. 10.1016/S0168-0102(02)00027-5 12067746

[36] Groenewegen HJ Berendse HW. Connections of the subthalamic nucleus with ventral striatopallidal parts of the basal ganglia in the rat. J Comp Neurol. (1990) 294:607–22. 10.1002/cne.902940408 2341628

[37] Takada M Tokuno H Hamada I Inase M Ito Y Imanishi M. Organization of inputs from cingulate motor areas to basal ganglia in macaque monkey. Eur J Neurosci. (2001) 14:1633–50. 10.1046/j.0953-816x.2001.01789.x 11860458

[38] Monakow KH Akert K Künzle H. Projections of the precentral motor cortex and other cortical areas of the frontal lobe to the subthalamic nucleus in the monkey. Exp Brain Res. (1978) 33:395–403. 10.1007/BF00235561 83239

[39] Carpenter MB Carleton SC Keller JT Conte P. Connections of the subthalamic nucleus in the monkey. Brain Res. (1981) 224:1–29. 10.1016/0006-8993(81)91113-6 7284825

[40] Canteras NS Shammah-Lagnado SJ Silva BA Ricardo JA. Afferent connections of the subthalamic nucleus: a combined retrograde and anterograde horseradish peroxidase study in the rat. Brain Res. (1990) 513:43–59. 10.1016/0006-8993(90)91087-W 2350684

[41] Lopes PSS Campos ACP Fonoff ET Britto LRG Pagano RL. Motor cortex and pain control: exploring the descending relay analgesic pathways and spinal nociceptive neurons in healthy conscious rats. Behav Brain Funct. (2019) 15:5. 10.1186/s12993-019-0156-0 30909927 PMC6432755

[42] Pagano RL Fonoff ET Dale CS Ballester G Teixeira MJ Britto LRG. Motor cortex stimulation inhibits thalamic sensory neurons and enhances activity of PAG neurons: possible pathways for antinociception. Pain. (2012) 153:2359–69. 10.1016/j.pain.2012.08.002 23017297

[43] Peyron R Faillenot I Mertens P Laurent B Garcia-Larrea L. Motor cortex stimulation in neuropathic pain. Correlations between analgesic effect and hemodynamic changes in the brain. A PET study. Neuroimage. (2007) 34:310–21. 10.1016/j.neuroimage.2006.08.037 17055297

[44] Nahmias F Debes C de Andrade DC Mhalla A Bouhassira D. Diffuse analgesic effects of unilateral repetitive transcranial magnetic stimulation (rTMS) in healthy volunteers. Pain. (2009) 147:224–32. 10.1016/j.pain.2009.09.016 19822394

[45] de Andrade DC Mhalla A Adam F Texeira MJ Bouhassira D. Neuropharmacological basis of rTMS-induced analgesia: the role of endogenous opioids. Pain. (2011) 152:320–6. 10.1016/j.pain.2010.10.032 21146300

[46] Ciampi de Andrade D Mhalla A Adam F Texeira MJ Bouhassira D. Repetitive transcranial magnetic stimulation induced analgesia depends on N-methyl-D-aspartate glutamate receptors. Pain. (2014) 155:598–605. 10.1016/j.pain.2013.12.022 24342462

