# Supplementary material for: Connectivity Patterns of Subthalamic Stimulation Influence Pain Outcomes in Parkinson's Disease
Source: Front Neurol. 2020 Feb 12;11:9. doi: 10.3389/fneur.2020.00009 (PMC7028764; doi:10.3389/fneur.2020.00009)
Supplement: Supplementary file 1 [file Data_Sheet_1.pdf]

Supplementary Material

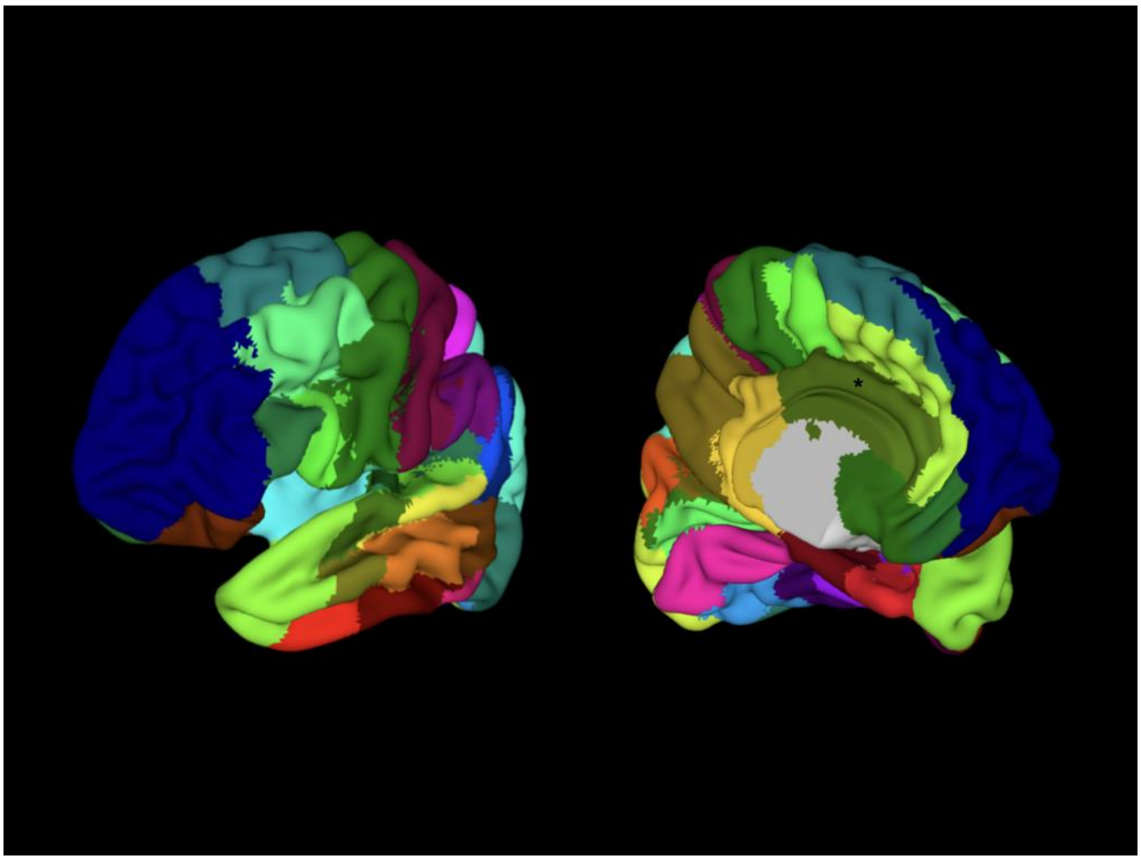

Supplementary Figure 1. Brain parcellations for connectivity correlations according to the human Harvard-Oxford atlas. Dark blue = prefrontal cortex (frontal pole); light blue = insular cortex; purple = post central gyrus; dark green = cingulate gyrus anterior division (*).

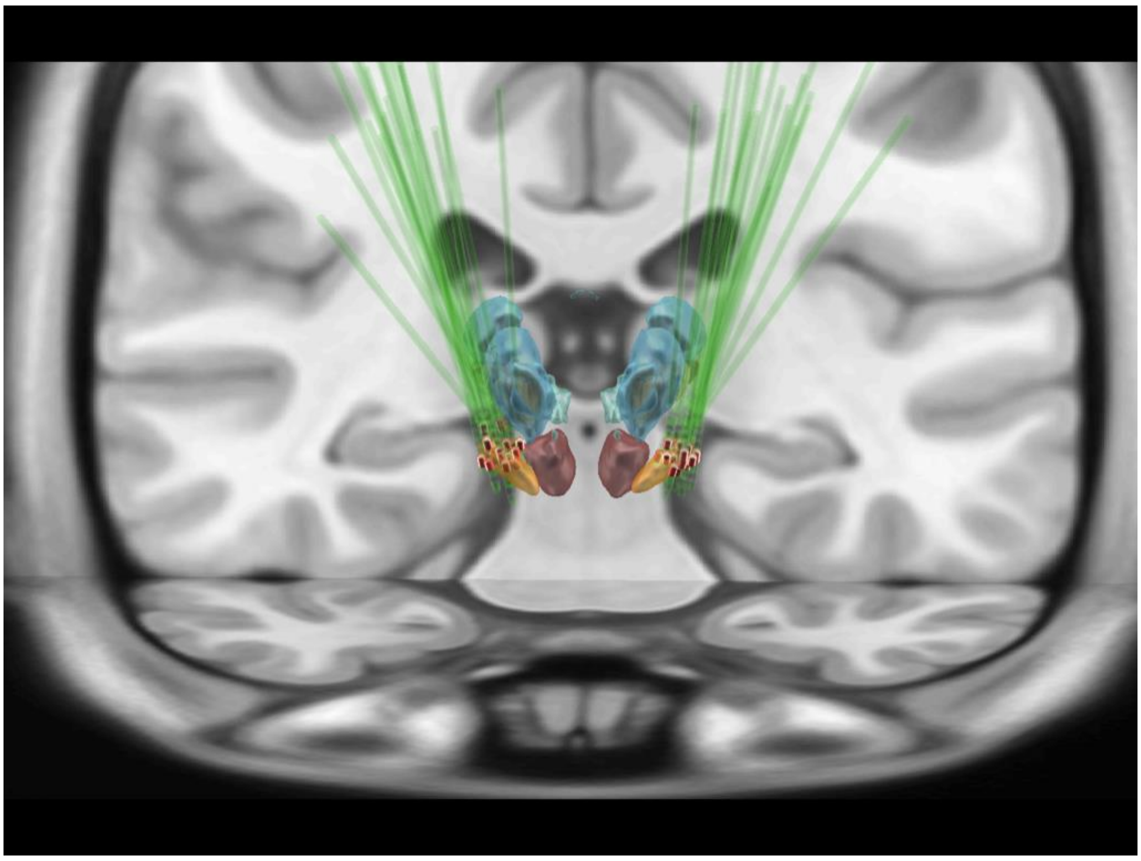

Supplementary Figure 2. Coronal view of the lead placement and the active contact (red highlighted) from all patients with pain before surgery. Orange = STN, red = red nucleus.
